# Synthesis of 1,2,3-Triazole Derivatives and Evaluation of their Anticancer Activity

**DOI:** 10.3797/scipharm.1302-04

**Published:** 2013-04-14

**Authors:** Nazariy Pokhodylo, Olga Shyyka, Vasyl Matiychuk

**Affiliations:** Ivan Franko National University of Lviv, Kyryla & Mefodiya Str., 6, 79005 Lviv, Ukraine.

**Keywords:** 1,2,3-Triazoles, Pfitzinger reaction, Anticancer activity, COMPARE analysis

## Abstract

Anticancer screening of several 1,2,3-triazoles with heterocyclic fragments has been performed. The 1,2,3-triazole derivatives were synthesized from available starting materials according to convenient synthetic procedures. The antitumor activity of the synthesized compounds was tested *in vitro* by the National Cancer Institute in NCI60 cell lines. It was observed that some compounds showed slight anticancer activity. One of them possessed a moderate activity against melanoma, colon, and breast cancer. Standard COMPARE analysis was performed at the GI_50_ level.

## Introduction

Triazoles and their derivatives are of great importance in medicinal chemistry and can be used for the synthesis of numerous heterocyclic compounds with different biological activities such as antiviral, antibacterial, antifungal, antituberculosis, anticonvulsant, antidepressant, anti-inflammatory, anticancer activities, etc [1]. They have been reported to be inhibitors of glycogen synthase kinase-3 [2], antagonists of GABA receptors [3, 4], agonists of muscarine receptors [5], be neuroleptic [6], and these compounds also show anti-HIV-1 [7], cytotoxic [8], antihistaminic [9], and antiproliferative activities [10]. Thus, the design and synthesis of novel triazole derivatives are the prospective direction of medicinal chemistry for the scientists working in this field.

Herein we describe the synthesis and anticancer activity of 1,2,3-triazoles containing some heterocyclic cores. The structures shown in the article were preselected from a number of substructure molecules by computer simulation and the most active of their represen-tatives were tested.

## Results and Discussion

### Chemistry

The starting materials, 1-aryl-5-methyl-1*H*-1,2,3-triazole-4-carboxylic acids **3a–e** were prepared by the reaction of aryl azides **1a–e** with ethyl acetoacetate **2** (Scheme 1). The produced acids **3a–e** were transformed to their corresponding acid chlorides **4a–e** by the action of SOCl_2_ and were used for the synthesis of oxadiazoles and flavonoids connected with the triazole core. Previously [11, 12], we found that 1-aryl-5-methyl-1*H*-1,2,3-triazole-4-carbonyl chlorides **4b,c** reacted with 5-substituted tetrazoles **5a,b** to give in all cases the corresponding 1,3,4-oxadiazoles **6a,b** containing a triazole substituent in the 5-position. The reactions started at approximately 60°C and were complete in 30 min to yield compounds **6a,b**. Initial tetrazoles **5a,b** were obtained by 1,3-dipolar cycloaddition of sodium azide to nitriles in the presence of ammonium chloride as a phase-transfer catalysis.

Compounds containing triazole and flavonoid fragments in one molecule were prepared by a three-step synthetic route. First, by the acylation of 1-(2-hydroxy-5-methylphenyl) ethanone **7** with the 1-phenyl-5-methyl-1*H*-1,2,3-triazole-4-carboxylic acid chloride **4a**. The resulting ester **8** in the presence of base underwent the Baker–Venkataraman rearrange-ment to yield 1,3-diketone **9**. The presence of the 1,3-diketone group and OH group in ortho-position in the arene ring allowed the acid catalysis heterocyclization to form compound **10** with a 56% general yield.

Previously, we have demonstrated [13] methods for the synthesis of 1-[5-methyl-1-(R-phenyl)-1*H-*1,2,3-triazol-4-yl]ethanones **12** from available azides **1** and acetylacetone and their use in the Pfizinger reaction with isatine **13** to yield 2-[1-(4-R-phenyl)-5-methyl-1*H-*1,2,3-triazol-4-yl]-4-quinoline-4-carboxylic acid **14**. Therein, we have shown the preparation of 2-[4-(4-R-5-methyl-1*H-*1,2,3-triazol-1-yl)phenyl]-4-quinolinecarboxylic acids **15** by the reaction of 1-[4-(4-R-5-methyl-1*H-*1,2,3-triazol-1-yl)phenyl] ethanones **3,21a**.

Moreover, the new activated ketomethylenic compounds β-nitrilesulfones **17**[14] and 1-(5-(R-amino)-1,2,4-thiadiazol-3-yl)propan-2-ones **18**[15] were used for 1,2,3-triazole synthesis in the Dimroth cyclization. The triazoles **19** and **20** were formed as mentioned earlier by the reaction of arylazides in sodium methoxide in methanol solution. It was found out that the corresponding 1,2,3-triazoles **19** and **20** were formed immediately after mixing the reagents at room temperature and precipitated in good yields from the reaction medium. Finally, compounds **21** were obtained by the reaction of azides with 1-(triphenyl-phosphoranylidene)acetone **16**. Acid chlorides **22** were used for the synthesis of amides **25** and oxadiazoles **26** from amine **23** and tetrazole **24,** respectively. Tetrazole **24** was readily prepared from the 3-cyanopyridines reaction with sodium azide in DMF in the presence of ammonium chloride [12].

### Biological activity

The main focus of the biological activity studies was on the search for compounds with antitumor activity. The newly synthesized compounds were selected by the National Cancer Institute (NCI) within the Developmental Therapeutic Program (www.dtp.nci.nih.gov) for *in vitro* cell line screening. Anticancer assays were performed according to the US NCI protocol, which was described elsewhere [16–20]. The compounds were first evaluated at one dose of the primary anticancer assay towards approximately 60 cell lines (concentration 10^−5^ M). The human tumor cell lines represent all forms of cancer (such as non-small-cell lung cancer, colon cancer, breast cancer, ovarian cancer, leukemia, renal cancer, melanoma, prostate cancer). In the screening protocol, each cell line was inoculated and pre-incubated for 24–48 h on a microtiter plate. Test agents were then added at a single concentration and the culture was incubated for an additional 48 h. The endpoint determinations were made with a protein binding dye, sulforhodamine B (SRB). The results for each test agent were reported as the percent growth of the treated cells compared to the untreated control cells. The preliminary screening results are shown in Table 1. The results for each compound are reported as the percent growth (GP). Range of growth (%) shows the lowest and the highest growth that was found among different cancer cell lines.

The synthesized 1,2,3 triazoles displayed slight **15a, 6b, 25** or low activity in the *in vitro* screen on the tested cell lines. However, there was a selective influence observed in some of the compounds on several cancer cell lines (Table 1). The compound **25** was highly active on the leukemia K-562 cell line (GP = 21.47%) and melanoma SK-MEL-5 cell line (GP = 23.91%). Compound **6b** was quite active on the leukemia SR cell line (GP = 65.29%) and compound **15a** on the renal cancer UO-31 cell line (GP = 65.29%). The majority of tested compounds displayed growth inhibition on the renal cancer cell line UO-31 and different cell lines of breast cancer and leukemia.

Finally, compound **25** was selected for *in vitro* testing against a full panel of about 60 tumor cell lines at 10-fold dilutions of five concentrations (100 μM, 10 μM, 1 μM, 0.1 μM, and 0.01 μM). Based on the cytotoxicity assays, three antitumor activity dose–response parameters were calculated for each experimental agent against each cell line: GI_50_ – molar concentration of the compound that inhibits 50% net cell growth; TGI – molar concentration of the compound leading to total inhibition; and LC_50_ – molar concentration of the compound leading to 50% net cell death. Values were calculated for each of these parameters if the level of activity was reached; however, if the effect was not reached or was exceeded, the value was expressed as greater or less than the maximum or minimum concentration tested. Mean graph midpoints (MG_MID) were calculated for each of the parameters, giving an averaged activity parameter over all cell lines for each compound. For the calculation of the MG_MID, insensitive cell lines were included with the highest concentration tested.

The most potent inhibition of human tumor cells was found for compound **25** (Table 2) (MG_MID GI50 −4.63 and −4.00, respectively).

The tested compound showed a broad spectrum of growth inhibition activity against human tumor cells, as well as some distinctive patterns of selectivity. In general, compound **25** selectively inhibited the growth of the colon cancer cell lines. We found that compound **25** possessed moderate activity on the breast cancer cell lines MDA-MB-468 and BT-549 (Log GI_50_ = −5.70, Log GI_50_ = −5.40), ovarian cancer cell lines OVCAR-4 (Log GI_50_ = −5.52), and melanoma cell line SK-MEL-5 (Log GI_50_ = −5.55). The most potent and selective cytotoxic activities against separate tumor cell lines are shown in Table 3.

It was found that 1,2,3-triazoles with the thiazole ring are quite active against tumor cell lines. It should be noted that compounds with the thiazole fragment directly bound to the 1,2,3-triazole core were not selected for the second stage of investigation in the NCI. On the contrary, 1,2,3-triazole amides with the thiazole moiety possessed moderate activity, among which compound **25** was the most active. Nowadays, new examples of such compounds are being synthesized and tested in the NCI.

The analysis of the activity of the triazole derivatives **6a**, **6b** allowed us to conclude that the presence of the methoxy group in the 1,3,4-oxadiazole fragment increased the anticancer activity on SR cell line (leukemia) up to 20%.

The combination of both 1,2,3-triazole and quinoline rings in one molecule resulted in interesting antitumor activity. However, compounds with the quinoline ring, bound directly to the 1,2,3-triazole core, were not selected for further investigation at the NCI at all or possessed low anticancer activity. On the contrary, compounds **15a**, **15b** were more active. In the case of compound **15a,** removal of the carboxyl group in the 1,2,3-triazole fragment led to the increase in the antitumor activity against the UO-31 cell line (renal cancer) up to 20%.

### COMPARE analysis

NCI’s COMPARE algorithm [21–24] allows the supposition of the biochemical mechanisms of action of novel compounds on the basis of their *in vitro* activity profiles when comparing with those of standard agents. Similarity of pattern to that of the seed is expressed quantitatively as a Pearson correlation coefficient (PCC). The results obtained with the COMPARE algorithm indicate that compounds high in this ranking may possess a mechanism of action similar to that of the seed compound. We used an accessible online tool – NCI COMPARE analysis to discover the similarity of compound **25** to the seed one (Table 4). Correlations with a PCC > 0,6 were selected as significant. Standard COMPARE analysis was performed at the GI_50_ level. Compound **25** did not yield any significant activity correlation with any standard agents. The obtained correlation coefficients didn’t allow a distinction between cytotoxicity mechanisms of the tested compounds with a high probability. Nevertheless, the compound showed moderate correlation with 4-ipomeanol (NSC: S349438). This may indicate that it has a unique mode of anticancer action.

## Experimental

All melting points were determined in capillary tubes in a Thiele apparatus and are uncorrected. The ^1^H NMR spectra were recorded on a Varian Mercury 400 instrument (400 MHz for ^1^H) and Bruker 500 (500 MHz for ^1^H, 125 MHz for ^13^C) with TMS or deuterated solvent as an internal reference. The mass spectra were run using the Agilent 1100 series LC/MSD and API-ES/APCI ionization mode. Satisfactory elemental analyses were obtained for the new compounds (C±0.17, H±0.15, N±0.12).

### General procedure for 1,3,4-oxadiazoles 6a,b and 26

Acid chloride **4** or **22**, 15 mmol, was added to the solution of 15 mmol of tetrazole **5a,b** or **24** in 15 mL of pyridine. The mixture was heated until nitrogen no longer evolved, then heated for 30 min under reflux, cooled, and diluted with 50 mL of water. The precipitate was filtered off, washed on a filter with water (up to 50 mL), dried in air, and purified by recrystallization with ethanol.

#### 2-(4-Methoxyphenyl)-5-[5-methyl-1-(3-methylphenyl)-1H-1,2,3-triazol-4-yl]-1,3,4-oxadiazole **6b**

Yield: 83%, mp 213–214°C (Ethanol). ^1^H NMR (400 MHz, DMSO-*d_6_*): 2.48 (s, 3H, CH_3_), 2.70 (s, 3H, CH_3_), 3.88 (s, 3H, CH_3_O), 7.09 (d, *J* = 8.4 Hz, 2H, H_Ar_-3,5), 7.39–7.48 (m, 3H, H_Ar_-2,4,6), 7.52 (t, *J* = 7.6 Hz, 1H, H_Ar_-5), 8.05 (d, *J* = 8.4 Hz, 2H, H_Ar_-2,6). ^13^C NMR (100 MHz, DMSO-*d_6_*) δ 163.7 (C), 162.4 (C), 158.1 (C), 139.8 (C), 135.9 (C), 135.4 (C), 131.4 (C), 130.8 (CH), 129.6 (CH), 128.7 (2xCH), 125.8 (CH), 122.4 (CH), 115.9 (C), 114.9 (2xCH), 56.2 (CH_3_O), 21.4 (CH_3_), 10.1 (CH_3_). MS *m/z* 348 (M+H)+. Anal. Calcd for C_19_H_17_N_5_O_2_, %: C, 65.69; H, 4.93; N, 20.16. Found, % C, 65.79; H, 4.81; N, 20.11.

#### 3-{5-[4-(5-Methyl-1H-1,2,3-triazol-1-yl)phenyl]-1,3,4-oxadiazol-2-yl}pyridine **26**

Yield: 93%, mp 188–189°C (Ethanol). ^1^H NMR (400 MHz, DMSO-*d_6_*): 2.47 s (3H, CH_3_), 7.61 (s, 1H, triazole), 7.66 (dd, *J* = 7.5, 2.8 Hz, 1H, H_Py_-5), 7.87 (d, *J* = 8.0 Hz, 2H, H_Ar_), 8.38 (d, *J* = 8.0 Hz, 2H, H_Ar_), 8.50 (d, *J* = 7.5 Hz, 1H, H_Py_-4), 8.79 (d, *J* = 2.8 Hz, 1H, H_Py_-6), 9.33 (s, 1H, H_Py_-2). ^13^C NMR (100 MHz, DMSO-*d_6_*) δ 164.0 (C), 162.8 (C), 152.7 (CH), 147.7 (CH), 139.1 (C), 134.4 (CH), 133.8 (C), 133.6 (CH), 128.4 (2xCH), 125.5 (2xCH), 124.5 (CH), 124.0 (C), 120.2 (C), 9.6 (CH_3_). MS *m/z* 305 (M+H)+. Anal. Calcd for C_16_H_12_N_6_O, %: C, 63.15; H, 3.97; N, 27.62. Found, % C, 63.11; H, 3.91; N, 27.72.

### Synthesis of 6-Methyl-2-(5-methyl-1-phenyl-1H-1,2,3-triazol-4-yl)-4H-chromen-4-one (10)

1-(2-Hydroxy-5-methylphenyl)ethanone (**7**) 1.5 g (0.01 mole) was dissolved in 5 mL of pyridine and triazole acid chloride **4a** 2.21 g (0.01 mole) was added, heated to 100 ° C and left to cool for 30 min at room temperature. Then the reaction mixture was poured into a mixture of 10 g of ice and 20 mL of 1M hydrochloric acid. The precipitate was filtered and crystallized from alcohol. Yield 76%. m.p. = 107–108 °C. Ester **8** 2.37 g (0.007 mole) was dissolved in 3 mL of pyridine at 50 °C. The mixture was added to a solution of potassium hydroxide 0.55 g (0.01 mole) and maintained for 1 h at 50 °C under stirring until the mixture became a homogeneous dense mass. The reaction mixture was poured into cool ice water and 10% solution of acetic acid. The precipitate was filtered and crystallized from alcohol. Yield 70%. m.p. = 59–60 ° C. To a suspension of diketone **9** 0.27 g (0.0008 mole) in 1 mL of glacial acetic acid the concentrated sulfuric acid 0.08 g was added and heated under reflux for 1 h. After cooling to room temperature the reaction mixture was poured into 15 g of ice and left for 30 min. Precipitate was filtered and crystallized with alcohol. Yield: 67%, mp 203–203°C (Ethanol). ^1^H NMR (400 MHz, DMSO-*d_6_*): 2.46 (s, 3H, CH_3_), 2.68 (s, 3H, CH_3_), 6.86 (s, 1H, H*_chromenone_*-2), 7.54–7.68 m (7H, H_Ph_+H*_chromenone-_*7,8), 7.83 (s, 1H, H*_chromenone_-*5). ^13^C NMR (100 MHz, DMSO-*d_6_*) δ 176.63 (CO), 158.25 (C), 154.33 (C), 137.43 (C), 135.82 (C), 135.46 (C), 135.35 (CH), 135.06 (C), 130.49 (CH), 130.20 (2xCH), 126.04 (2xCH), 125.18 (CH), 124.24 (C), 118.45 (CH), 107.43 (CH), 21.31 (CH_3_), 10.79 (CH_3_). MS *m/z* 318 (M+H)+. Anal. Calcd for C_19_N_15_N_3_O_2_, %: C, 71.91; H, 4.76; N, 13.24. Found, % C, 71.84; H,4.86; N, 13.29.

### Synthesis of 2-[1-(4-Fluorophenyl)-5-methyl-1H-1,2,3-triazol-4-yl]quinoline-4-carboxylic acid (14a)

Isatine (**13**) 1.47 g (0.01 mol) was dissolved in 25 mL of 8 M solution of KOH, then ketone **12a** 2.21 g (0.01 mol) and ethanol were added until the mixture became homogenous. The mixture was refluxed for 2 h, then cooled and 10 mL of water was added. The mixture was acidified with AcOH to pH ≈ 6–7 and the solid was filtered. The products were recrystallized with ethanol. Yield 88%, mp >300°C. ^1^H NMR (400 MHz, DMSO-*d_6_*): 2.50 (s, 3H, CH_3_), 7.41 (t, 2H, *J* = 8.5 Hz, H_Ar_-3,5). 7.63 (t, 1H, *J* = 7.6 Hz, H_quinoline_-7), 7.71 (dd, 2H, *J* = 8.5, 4.7 Hz, H_Ar_-2,6), 7.64 (s, 1H, H_quinoline_-6), 7.73–7.81 (m, 1H), 8.08 (d, *J* = 8.5 Hz, 1H, H_quinoline_-5), 8.76–8.85 (m, 2H, H_quinoline_-3,8), 13.55 br.s (1H, COOH). ^13^C NMR (100 MHz, DMSO-*d_6_*) δ 167.9 (CO), 163.1 (d, *J* = 249.1 Hz, C-F), 152.0 (C), 148.9 (C), 142.7 (C), 137.2 (C), 134.8 (C), 132.6 (C), 130.1 (CH), 130.0 (CH), 128.2 (d, *J* = 9.0 Hz, 2xCH), 127.8 (CH), 126.4 (CH), 124.3 (C), 120.8 (CH), 117.1 (d, *J* = 23.1 Hz, 2xCH), 11.1 (CH_3_). Calculated, %: C 68.91; H 4.14; N 16.84. C_19_H_14_N_4_O_2_. Found, %: C 69.08; H 4.27; N 16.96.

### N-(5-Benzyl-1,3-thiazol-2-yl)-4-(5-methyl-1H-1,2,3-triazol-1-yl)benzamide (25)

The mixture of 4-azidobenzoic acid **1b** 1.63 g (0.01 mole) and 1-(triphenylphosphoran-ylidene) acetone 3.18 g (0.01 mole) of and 10 mL of benzene was heated for 5 h. Benzene was evaporated under reduced pressure. The dry residue was dissolved in a 5% solution of NaOH. Insoluble triphenylphosphine oxide was filtered off, and the filtrate was acidified with concentrated HCl. The formed precipitate was filtered and purified by recrystallization with ethanol. The yield of 4-(5-Methyl-1H-1,2,3-triazole-1-yl) benzoic acid **21b was** 82%. m.p. 243–244 °C. ^1^H NMR (400 MHz, DMSO-*d_6_*): 2.41 (s, 3H, CH_3_), 7.60 (s, 1H, triazole), 7.69 (d, 2H, *J* = 8,1, 2,6-H_Ph_), 8,15 (d, 2H, *J* = 8,1, 3,5-H_Ph_). The mixture of acid **21b** 1 g (0.05 mole) and 0.37 mL (0.005 mole) thionyl chloride in 25 mL of dioxane was heated under reflux until gas evolution ceased. The mixture was cooled and the precipitate was filtered. The yield of chloride **22** was 84%, m.p. 138 °C. 5-Benzyl-1,3-thiazol-2-amine **23** 0.47 g (5 mmol) was dissolved in 15 mL of dioxane and added to 0.7 mL (5 mmol) of triethylamine followed by the 4-(5-Methyl-1H-1,2,3-triazole-1-yl) benzoic acid chloride **22** 1.11 g (5 mmol). The reaction mixture was heated to reflux and maintained for 1 h at room temperature. The mixture was diluted with water and the solid was filtered. Yield 88%, mp 231–232°C. ^1^H NMR (400 MHz, DMSO-*d_6_*): 2.42 (s, 3H, CH_3_), 4.12 (s, 2H, CH_2_), 7.17–7.36 (m, 6H), 7.61 (s, 1H, H_triazole_), 7.72 (d, *J* = 8.6 Hz, 2H, H_Ar_-2,6), 8.29 (d, *J* = 8.6 Hz, 2H, H_Ar_-3,5), 12.60 (s, 1H, NH). ^13^C NMR (100 MHz, DMSO-*d_6_*) δ 163.9 (CO), 158.4 (C), 140.1 (C), 139.2 (CH), 134.4 (C), 133.7 (C), 133.5 (CH), 133.1 (C), 131.6 (C), 129.8 (2xCH), 128.7 (2xCH), 128.6 (2xCH), 126.6 (CH), 124.4 (2xCH), 32.7 (CH_2_), 9.5 (CH_3_). MS *m/z* 378 (M+H)+. Anal. Calculated, %: C 63.98; H 4.56; N 18.65. C_20_H_17_N_5_OS. Found, %: C 63.87; H 4.71; N 18.74.

### Cytotoxic activity against malignant human tumor cells

A primary anticancer assay was performed on a panel of approximately 60 human tumor cell lines derived from nine neoplastic diseases, in accordance with the protocol of the Drug Evaluation Branch, National Cancer Institute, Bethesda. The tested compounds were added to the culture at a single concentration (10^−5^ M) and the cultures were incubated for 48 h. Endpoint determinations were made with a protein binding dye, sulforhodamine B (SRB). Results for each tested compound were reported as the percent growth of the treated cells when compared to the untreated control cells. The percent growth was evaluated spectrophotometrically versus controls not treated with the test agents. The cytotoxic and/or growth inhibitory effects of the most active selected compounds were tested *in vitro* against the full panel of about 60 human tumor cell lines at 10-fold dilutions of five concentrations ranging from 10^−4^ to 10^−8^ M. The 48-h continuous drug exposure protocol was followed and an SRB protein assay was used to estimate cell viability or growth.

Using the seven absorbance measurements [time zero, (T_z_), control growth in the absence of drug, (C), and test growth in the presence of drug at the five concentration levels (T_i_)], the percent growth was calculated at each of the drug concentrations levels. Percent growth inhibition was calculated as:

$$\begin{array}{l} {}[({\text{T}}_{\text{i}}-{\text{T}}_{\text{z}})/(\text{C}-{\text{T}}_{\text{z}})]\times 100\;\text{for concentrations for which }{\text{T}}_{\text{i}}\geq {\text{T}}_{\text{z}}\\ {}[({\text{T}}_{\text{i}}-{\text{T}}_{\text{z}})/{\text{T}}_{\text{z}}]\times 100\;\text{for concentrations for which }{\text{T}}_{\text{i}}<{\text{T}}_{\text{z}}. \end{array}$$

Three dose-response parameters were calculated for each compound. Growth inhibition of 50% (GI_50_) was calculated from [(T_i_ − T_z_)/(C − T_z_)] × 100 − 50, which is the drug concentration resulting in a 50% lower net protein increase in the treated cells (measured by SRB staining) as compared to the net protein increase seen in the control cells. The drug concentration resulting in total growth inhibition (TGI) was calculated from T_i_ = T_z_. The LC_50_ (concentration of drug resulting in a 50% reduction in the measured protein at the end of the drug treatment as compared to that at the beginning) indicating a net loss of cells following treatment was calculated from [(T_i_ − T_z_)/T_z_] × 100 = −50. Values were calculated for each of these three parameters if the level of activity was reached; however, if the effect was not reached or was exceeded, the value for that parameter was expressed as more or less than the maximum or minimum concentration tested. The logGI_50_, logTGI, logLC_50_ were then determined, defined as the mean of the logs of the individual GI_50_, TGI, LC_50_ values. The lowest values were obtained with the most sensitive cell lines. Compounds having these values ≤ 4 were declared to be active.

## Figures and Tables

**Sch. 1:**
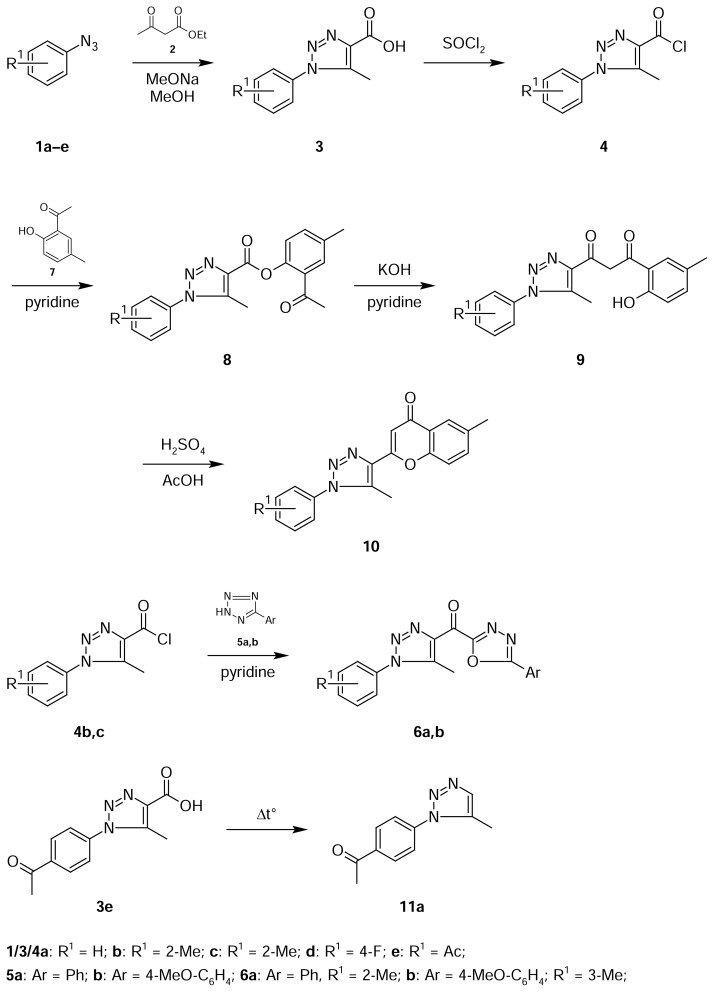
Preparation of flavanoic and oxadiazole derivatives of 1,2,3-triazoles.

**Sch. 2:**
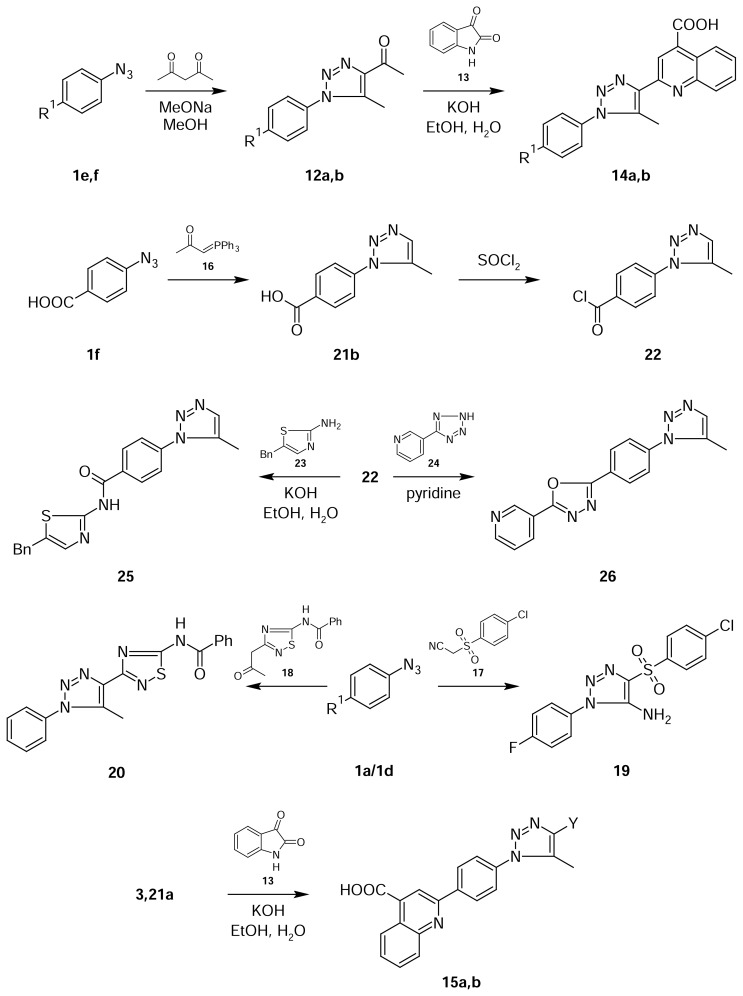
Synthesis of quinolone, 1,2,4-thiadiazole and 1,3-thiazole derivatives of 1,2,3-triazoles.

**Sch. 3:**
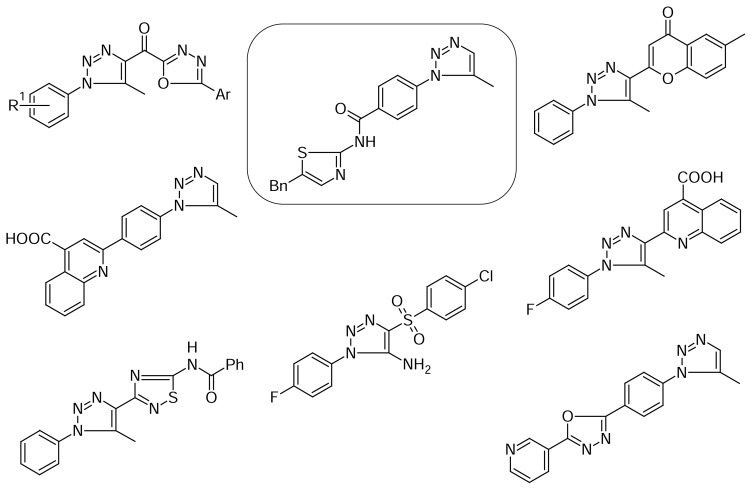
The most active anticancer heterocyclic derivatives of 1,2,3-triazoles.

**Tab. 1:** Anticancer activity screening at one dose assay (10^−5^ M)

Comp.	Mean growth, %	Range of growth, %	The most sensitive cell lines, (growth %)
**6a [11]**	104.97	75.66 to 155.37	Colon cancer: HCT-15 (75.66) Leukemia: RPMI-8226 (79.56), SR (86.45) Renal cancer: UO-31 (86.48)
**6b**	98.68	65.29 to 156.38	Leukemia: SR (65.29), RPMI-8226 (69.42) Non-small cell lung cancer: HOP-92 (72.57), NCI-H522 (78.53) Renal cancer: A498 (75.93) Breast cancer: MCF7 (83.24)
**10**	93.84	73.89 to 116.35	Renal cancer: UO-31 (73.89), 786-0 (75.92) Non-small cell lung cancer: HOP-92 (76.37) Leukemia: SR (77.19)
**14a**	105.21	78.72 to 145.87	Colon cancer: HCC-2998 (78.72) Renal cancer: UO-31 (85.15)
**15a [13]**	104.33	65.29 to 129.04	Renal cancer: UO-31 (65.29) Ovarian cancer: IGROV1 (71.28)
**15b [13]**	102.77	86.66 to 123.12	Breast cancer: HS 578T (86.66), MCF7 (88.96) Renal cancer: UO-31 (87.37) CNS cancer: SF-539 (89.57)
**19 [14]**	97.50	72.32 to 136.13	Renal cancer: UO-31 (72.32), ACHN (81.54) Leukemia: RPMI-8226 (82.49) Melanoma: UACC-62 (85.10) Non-small cell lung cancer: NCI-H522 (86.28)
**20 [15]**	106.14	83.34 to 162.47	Colon cancer: HCC-2998 (83.24) Leukemia: RPMI-8226 (85.87) Breast cancer: MCF7 (86.98)
**25**	65.87	21.47 to 103.47	Leukemia: K-562 (21.47), SR (22.54), MOLT-4 (33.38), RPMI-8226 (37.67) Melanoma: SK-MEL-5 (23.91) Breast cancer: MDA-MB-468 (28.28) Ovarian cancer: OVCAR-4 (33.03) Renal cancer: UO-31 (42.11)
**26**	109.88	92.25 to 183.68	Breast cancer: MCF7 (92.25) Leukemia: MOLT-4 (93.17)

**Tab. 2:** Summary of anticancer screening data at dose-dependent assay

Comp.	N	Log GI_50_			Log TGI		
		N_1_	Range	MG_MID	N_2_	Range	MG_MID
**25**	56	28	−5.70 to −4.29	−4.63	1	−4.13 to −4.00	−4.00

N-number of human tumor cell lines tested at the 2nd stage assay

N_i_-number of sensitive cell lines (parameters Log GI_50_ and Log TGI<−4.00)

**Tab. 3:** The influence of compound **25** on the growth of individual tumor cell lines

Compd.	Disease	Cell line	Log GI_50_
25	Colon cancer	KM12	−5.43
	Melanoma	SK-MEL-5	−5.55
	Melanoma	UACC-62	−5.48
	Ovarian cancer	OVCAR-4	−5.52
	Renal cancer	CAKI-1	−5.33
	Prostate cancer	PC-3	−5.37
	Breast cancer	BT-549	−5.40
	Breast cancer	MDA-MB-468	−5.70

**Tab. 4:** COMPARE analysis results for the tested compound

Comp.	PCC	Target	Target vector NSC	Count common cell lines	Seed StDev	Target StDev
**25**	0.424	4-ipomeanol	S349438	43	0.332	0.162
**Target mechanism of action**						

Ipomeanol is activated by mixed function oxidases in vivo to its epoxide form, an alkylating agent that covalently binds cell macromolecules. This agent causes cell death by a p53-independent mechanism.
